# Identification of circRNA CDR1as/miR-214-3p regulatory axis in Legg-Calvé-Perthes disease

**DOI:** 10.1186/s13023-024-03394-5

**Published:** 2024-10-15

**Authors:** Xia Lan, Ronghui Yu, Jianyun Xu

**Affiliations:** Orthopedic Hospital, The First Affiliated Hospital, Jiangxi Medical College, Nanchang University, No. 1519, Dongyue Avenue, Nanchang, Jiangxi Province 330006 P.R. China

**Keywords:** Legg-Calvé-Perthes disease, CircRNA CDR1as, MiR-214-3p, Macrophage polarization, Angiogenesis

## Abstract

**Background:**

Legg-Calvé-Perthes disease (LCPD) commonly occurs among adolescents, threatening their health. However, the potential mechanism underlying LCPD remains unclear. miR-214-3p is shown as a critical role in LCPD development with unspecified upstream regulators.

**Methods:**

Levels of miR-214-3p and circCDR1as in healthy controls and LCPD patients were determined by qRT-PCR. The role of circCDR1as/miR-214-3p axis in LCPD was determined by testing the cell viability and apoptosis in TC28 cells and primary chondrocytes. Regulation between circCDR1as and miR-214-3p was examined by RIP and ChIP assays. The inflammatory response and angiogenesis were evaluated by M2 macrophage polarization and HUVECs tumor formation.

**Results:**

circCDR1as was overexpressed in LCPD patients with a negative correlation with miR-214-3p. Inhibition of circCDR1as alleviated the cell viability and apoptosis of DEX-treated chondrocytes, stimulated M2 macrophage polarization and angiogenesis. miR-214-3p was proved as a downstream effector to participate in circCDR1as mediated actions. circCDR1as recruited PRC2 complex to epigenetically suppress miR-214-3p.

**Conclusion:**

Our study illustrated the role and mechanism of circCDR1as in LCPD development by targeting miR-214-3p, highlighting its potential in the therapy for LCPD.

**Supplementary Information:**

The online version contains supplementary material available at 10.1186/s13023-024-03394-5.

## Introduction

Legg-Calvé-Perthes disease (LCPD) is a common pediatric disease of femoral head osteonecrosis that mainly occurs among children between 2 and 12 years old, which is characterized by avascular necrosis of the femoral head and deformities of the femoral head and acetabulum [1, 2]. Disruption of osteoblastic differentiation can lead to osteonecrosis, demonstrated by the downregulation of osteoblast-specific markers such as RUNX2 and COL1A1 in the osteonecrosis of femoral head [3]. The prognosis of LCPD in the early stage is optimistic, while the treatment for advanced LCPD only has poor outcomes [4]. Studies have demonstrated that LCPD is mainly caused by the uncoupling of bone metabolism [5, 6]. However, the exact pathophysiology of this disease remains unclear, and the discovery of its mechanism remains a challenge.

Circular RNAs (circRNAs) belong to noncoding RNAs that are characterized by covalently closed continuous loops with neither 5’-3’ polarity nor a polyadenylated tail [7]. CircRNAs are reported to regulate the expression of target genes by serving as miRNA sponges or interacting with other molecules [8]. Accordingly, circRNAs have been well characterized to play an important role in various cellular functions, such as tumorigenesis, inflammation, and apoptosis [9]. Previous studies have reported that abnormally elevated circCDR1as was related to the poor prognosis in different kinds of cancers [10–12]. What’s more, a recent report unveiled that circCDR1as could play an important role in the musculoskeletal system [13]. In particular, circCDR1as was proven to suppress osteogenic differentiation of bone marrow mesenchymal stem cells in steroid-induced osteonecrosis of the femoral head by regulating the miR-7-5p/WNT5B axis, and its expression in plasma and local tissues were closely associated with the disease severity in patients with osteonecrosis of the femoral head [14, 15]. However, whether circCDR1as could affect primary chondrocytes directly and its exact role in LCPD has not been investigated yet.

MicroRNAs (miRNAs) belong to a class of non-coding RNAs with a length of 18 ~ 22 nucleotides, inhibiting the expression of downstream targets by binding to the 3’-untranslated region (3’UTR) and inducing mRNA degradation or translational repression [16]. The abnormal expression of miRNAs could result in cellular and tissue disorders. Intriguingly, miRNAs have been widely studied in musculoskeletal-related diseases, such as osteoarthritis and tendon injuries [17, 18]. More importantly, several miRNAs have been shown to play significant roles in chondrogenesis and LCPD. For example, miR-206 has been found to promote cell apoptosis in LCPD via suppression of SRY-box 9 [19]. Moreover, overexpression of miR-214-3p has been demonstrated to inhibit chondrocyte differentiation by targeting ATF4 [20]. Additionally, miR-214-3p has been indicated to enhance chondrocyte viability and inhibit apoptosis via downregulation of Bax in LCPD. However, the upstream regulator of miR-214-3p in the development of LCPD remains unclear [21].

Macrophages are known to play a critical role in the chronic inflammatory repair processes [22, 23]. They can act as proinflammatory (further damaging tissue) or anti-inflammatory (repairing tissue) roles depending on the environment. The proinflammatory M1 phenotype is characterized by the expression of IL-1β, IL-6, IL-12β, CD68 and TNF-α, while the anti-inflammatory M2 phenotype is characterized by the expression of Arg1, IL-4 and IL-10 [24, 25]. The switching between the M1 and M2 phenotype of macrophages has been shown to exert a fundamental effect on the progression of LCPD [26]. An assessment of the role of miR-214-3p for macrophage polarization would reveal a potential mechanism for the inflammation responses in LCPD.

In this study, we first discovered that aberrantly upregulated circCDR1as in LCPD patients exerted a negative correlation with the levels of miR-214-3p, which was downregulated in LCPD patients. Functionally, inhibition of circCDR1as significantly alleviated cell viability and apoptosis of DEX-treated chondrocytes. Moreover, miR-214-3p was identified as a downstream target of circCDR1as, of which depletion abrogated the protective effects on chondrocytes from silencing of circCDR1as. On the molecular level, circCDR1as was found to recruit PRC2 complex into miR-214-3p promoter region and inhibit its expression by H3K27me3 modification. Silencing of circCDR1as in chondrocytes also proved to stimulate M2 macrophage polarization and angiogenesis by targeting miR-214-3p. Taken together, our study illustrated the mechanism of circCDR1as in LCPD development by suppressing miR-214-3p, highlighting the therapeutic potential of circCDR1as/miR-214-3p axis in the treatment of LCPD.

## Materials and methods

### Healthy controls and LCPD patients’ samples

All samples, from volunteer donors (healthy controls, volunteers under healthy conditions after amputation surgery due to severe injury, such as car crashing or falling from a building) and LCPD patients with similar ages (*n* = 20, < 14 years old) and the same sex (17% girl, 83% boy), were collected from The First Affiliated Hospital, Jiangxi Medical College, Nanchang University with the approval from the ethics committee of our hospital. Before treatment at the hospital, all patients were provided with written informed consent to allow any excess tissue for research studies. LCPD was diagnosed based on ultrasonographic examination and magnetic resonance imaging. Samples were snap-frozen in Optimal Cutting Temperature and stored at -80 °C until use. The pathological status of the specimens were provided by the hospital.

### Cell culture and transfection

Primary chondrocytes were isolated from LCPD and healthy control femoral head cartilage tissues via collagenase digestion of cartilage and cultured in monolayer according to a previous study [27]. Cells of the first passage were used in the experiments of the study. The human cartilage cell line TC28 and human umbilical vein endothelial cells (HUVECs) used in this study was purchased from the Shanghai Institute of Cell Biology (Shanghai, China). All cells were cultured with RPMI-1640 (Invitrogen, Carlsbad, CA, USA) containing 10% fetal bovine serum (FBS, Invitrogen) and penicillin-streptomycin solution (Invitrogen). The M2 macrophages were isolated from human peripheral blood mononuclear cells (PBMCs) by positive selection using CD14 microbeads (Miltenyi Biotec, Cologne, Germany), followed by 50 ng/mL recombinant G-CSF (Leukine, Genzyme Corporation, UK) treatment and 20 ng/mL IL-4 (Abcam, Cambridge, UK) stimulation. CD206 staining (Biolegend, San Diego, USA) was used to characterize M2 macrophages. Treatment of 0.01 mM dexamethasone (DEX, Sigma-Aldrich, MO, USA) for 2 h was applied to induce LCPD in vitro. miR-214-3p inhibitor and negative control (NC inhibitor) were purchased from Genepharma (Shanghai, China). Small interfering RNAs (siRNAs) targeting circCDR1as were designed and synthesized by RiboBio (Guangzhou, China). Cell transfections were performed by using Lipofectamine 2000 transfection reagent (cat. 11668019, Invitrogen) according to the manufacturer’s instructions. After 48 h, the cells were collected for subsequent experiments.

### RNA extraction and quantitative real-time PCR (qRT-PCR)

RNA Subcellular Isolation Kit (Cat. 25501, Active Motif, USA) was used to extract nuclear and cytoplasmic RNA respectively according to the manufacturer’s protocol. U6 was used for the internal control of nuclear RNA and GAPDH RNA was used for the internal control of cytoplasmic RNA. For the total RNA extraction, cells or tissues were dissolved in TRIzol reagent (Cat. 15596018, Invitrogen), and total RNA was obtained according to the manufacturer’s protocol. The RNA was then tested for quality and synthesized into cDNA using an iScript cDNA Synthesis Kit (Cat. 1708891, Bio-Rad, USA). qPCR was performed using SYBR Green Supermixes (Cat. 1708882, Bio-Rad). GAPDH and U6 were used as endogenous controls for normalization. Relative levels of RNA expressions were normalized and analyzed using the 2^−ΔΔCt^ method. All primers used in this study were listed in Table 1.

**Table 1:** The primer sequences used in qRT-PCR

Gene	Forward primer (5’-3’)	Reversed primer (5’-3’)
circCDR1as	ACCCAGTCTTCCATCAACTGG	ACCTTGACACAGGTGCCATC
EZH2	AATCAGAGTACATGCGACTGAGA	GCTGTATCCTTCGCTGTTTCC
miR-214-3p	GTGCAGGGTCCGAGGT	ATCATAGAGGAAAATCCACG
U6	GCTTCGGCAGCACATATACTAAAAT	CGCTTCACGAATTTGCGTGTCAT
GAPDH	AGGTCGGTGTGAACGGATTTG	TGTAGACCATGTAGTTGAGGTCA

### Western blot analysis

Cells washed with cold PBS or tissues after homogenization were incubated with lysis buffer on ice for 30 min. After centrifugation, the supernatant containing the lysate was collected and stored at -80 °C. A BCA assay kit (Cat. 5000001, Bio-Rad) was used to determine protein concentrations. Protein samples were denatured and then separated by SDS-PAGE and transferred to PVDF membranes (Cat. IPVH00010, Millipore, USA). After blocking with non-fat milk for 1 h, membranes were incubated overnight at 4 °C with the indicated primary antibodies from Cell Signaling Technology (Danvers, USA) at a 1:1000 dilution (Bax: #14796; Bcl-2: #15071; caspase3: #9662; cleaved-caspase3: #9664; β-Actin: #3700). After washing 3 times, the membranes were incubated with goat anti-rabbit or anti-mouse HRP-conjugated secondary antibody at a 1:5000 dilution (#7074 or #7076, Cell Signaling Technology). The signals were analyzed using an ECL detection kit (Cat. 32106, Pierce Biotechnology, USA).

### Cell viability assay

The cell counting kit-8 (CK04-11, Dojindo Molecular Technologies, MD, USA) was used to test cell viability. After the indicated treatment, 1 × 10^4^ cells were counted and seeded into 96-well plates. Before testing, 10 µl of the working solution was added to each well and cells were continuing cultured for 4 h. The absorbance at 450 nm was measured using a spectrophotometer.

### Cell apoptosis analysis by flow cytometry

To measure the level of cell apoptosis, 2 × 10^5^ cells were collected and washed 3 times by PBS and then dissolved in 500 µL of annexin V binding buffer, followed by the staining with 5 µL of annexin V for 10 min and 5 µL of Propidium Iodide (PI) for 15 min. The stained cells were then detected and analyzed by flow cytometry in the guidance of the manufacturer’s instructions.

### RNA immunoprecipitation

RNA immunoprecipitation (RIP) was conducted using a Magna RIP RNA-binding protein immunoprecipitation (IP) kit (Millipore, Cambridge, MA, USA) in the guidance of the manufacturer’s protocols. The cells were collected and dissolved in IP lysis buffer, followed by mechanical shearing using a homogenizer. Antibodies against indicated proteins (EZH2: #ab191250; SUZ12: #ab12073; EED: #ab240650; GAPDH: #ab128915, Abcam) and negative control immunoglobulin G (IgG) (catalog no. 12–371; EMD Millipore) were then incubated with the cell lysis under 4 °C overnight. After washing 3 times, the mixture was added with magnetic beads with streptavidin coating and then incubated for 2 h. The extracted RNAs were then measured and analyzed by qRT-PCR.

### RNA pull-down

For the assay of RNA pull-down, the Biotin RNA Labeling Mix (Roche Diagnostic, Indianapolis, USA) and T7 RNA polymerase (Roche) were used to synthesize RNAs labeled with biotin in vitro. Cell nuclear extraction (2 µg) was isolated and incubated with 100 pmol biotin-labeled RNAs. 100 µL of streptavidin agarose beads were washed and added into each binding mixture, followed by incubation for 1 h under room temperature. After that, Beads were briefly washed three times and then denatured by boiling in 2X SDS buffer, and the eluted protein samples were separated and detected by standard western blot technique.

### Chromatin immunoprecipitation (ChIP) assay

ChIP assay was conducted by the EZ-ChIP Kit (Millipore, USA) by the protocol. Chromatin of cells was obtained and immunoprecipitated by antibody against EZH2 (#ab191250, Abcam) and H3K27me3 (#ab6002, Abcam). Antibody against IgG (Abcam, USA) was utilized as the negative control. Real-time PCR was used to calculate and determine the fold enrichment as the ratio of EZH2/IgG or H3K27me3/IgG.

### Tube formation assay

The matrix, formed from basement membrane extract (Geltrex), was layered into 12 wells of clear, polystyrene, flat-bottom well plates (Corning/Costar-Sigma, Taufkirchen; Germany). After coating, the wells were overlayed with 1 × 10^5^ cells/well with 400 µL of conditioned medium. Tube formation was checked during the first 10 h by microscope under bright light. The tube formation ability was assessed by the length of formed tubes calculated from 6 randomly taken pictures for each well after the indicated treatment.

### Statistical analysis

Statistical analysis was performed using GraphPad Prism 5. All experiments were conducted at least three times. Data were presented as the mean ± SD and analyzed by one-way ANOVA followed by Tukey’s multiple comparison test or Student’s t-test. *P* < 0.05 was considered statistically significant.

## Results

### Expression patterns of circCDR1as and mir-214-3p in LCPD

**Fig. 1:**
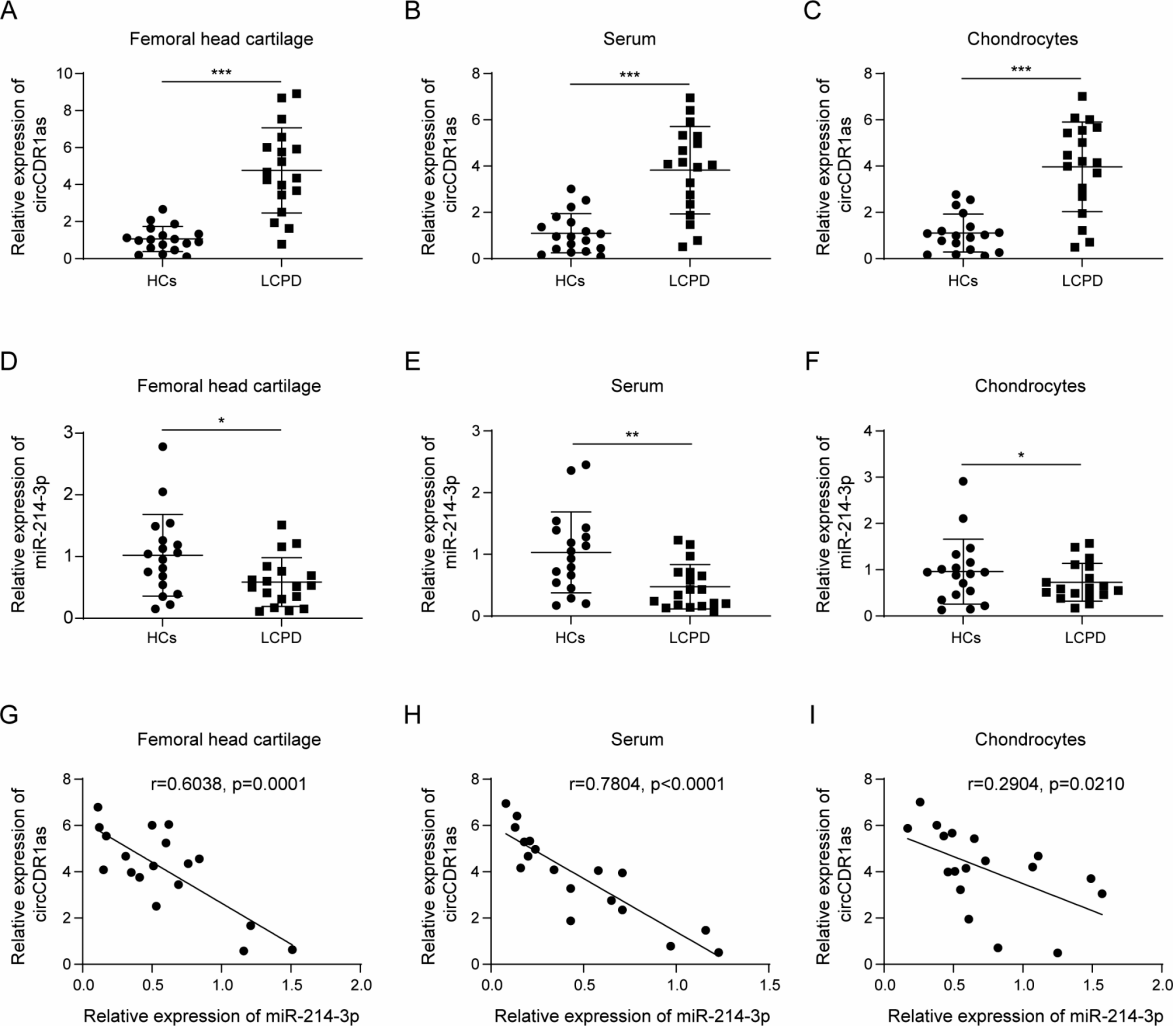
Expression patterns of circCDR1as and miR-214-3p in LCPD. Expression level of circCDR1as in (**A**) femoral head cartilage, (**B**) serum and (**C**) chondrocytes collected from healthy controls (HC) and LCPD patients, respectively. Expression level of miR-214-3p in femoral (**D**) head cartilage, (**E**) serum and (**F**) chondrocytes collected from healthy controls (HC) and LCPD patients, respectively. Correlation analysis between circCDR1as and miR-214-3p in femoral head (**G**) cartilage, (**H**) serum and (**I**) chondrocytes collected from LCPD patients. *N* = 18, *P < 0.05, **P < 0.01, ***P < 0.001

To determine the potential functions of circCDR1as and miR-214-3p in the development of LCPD, we first assessed their expression patterns between LCPD patients and healthy controls (HCs). As shown in Fig. 1, circCDR1as was found to be significantly upregulated in the femoral head cartilage (Fig. 1A), serum (Fig. 1B) and chondrocytes (Fig. 1C) that were collected from LCPD patients compared to those from HCs. On the other hand, the expression of miR-214-3p was obviously downregulated in the above samples from LCPD patients compared to those from HCs (Fig. 1D-F). To explore the relationship between the expression of circCDR1as and miR-214-3p, we further analyzed their expression in a large cohort of tissue samples from LCPD patients. Intriguingly, the level of circCDR1as was found to be negatively correlated with the expression of miR-214-3p in the femoral head cartilage (Fig. 1G), serum (Fig. 1H) and chondrocytes (Fig. 1I). Taken together, these results strongly indicated that circCDR1as was upregulated in LCPD patients, while miR-214-3p was downregulated; and their expression levels in LCPD patients were negatively correlated.

### Role of circCDR1as in TC28 cells and primary chondrocytes exposed with DEX

**Fig. 2:**
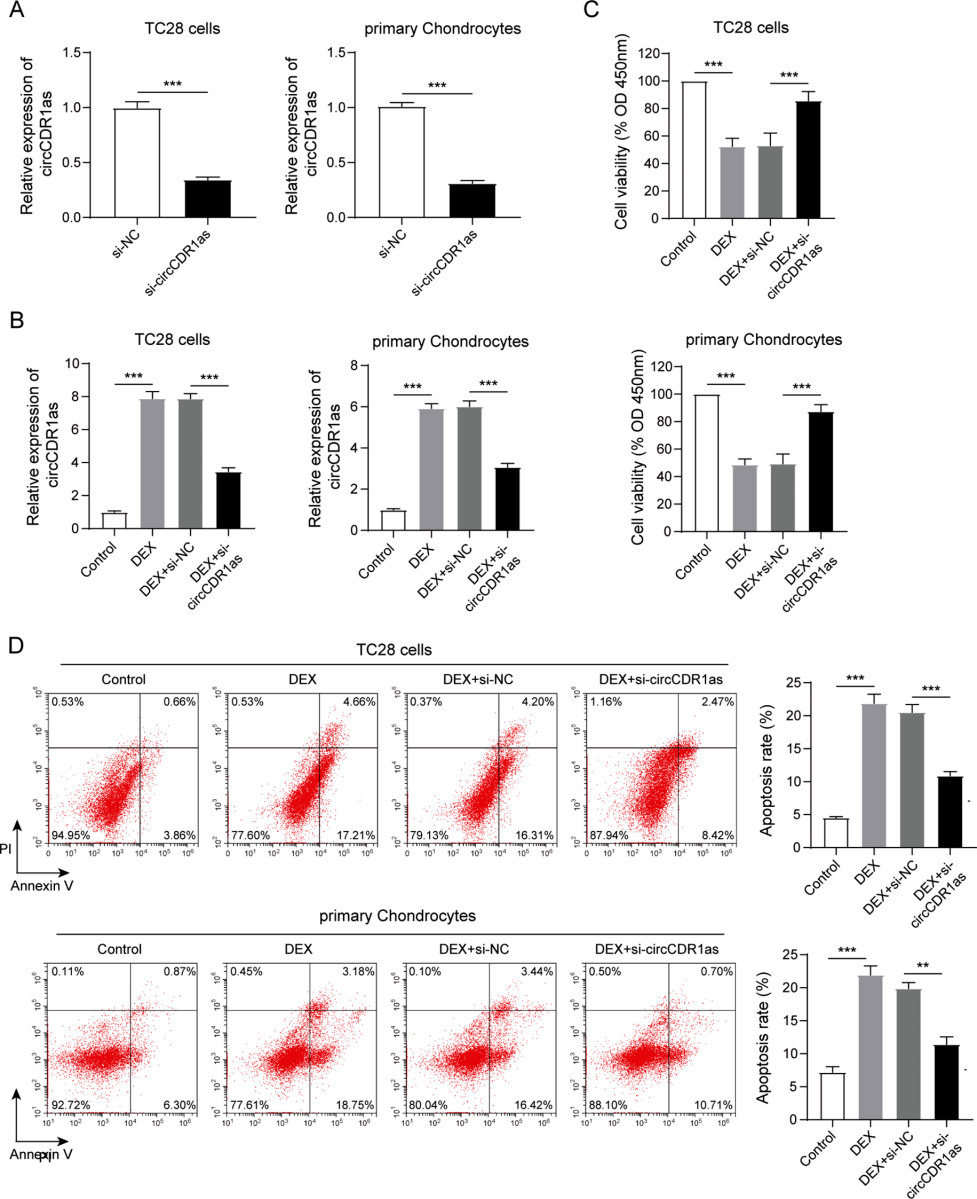
Role of circCDR1as in TC28 cells and primary chondrocytes exposed with DEX. (**A**) Determination of siRNA efficiency against circCDR1as. (**B**) Detection of circCDR1as expression in TC28 cells and primary chondrocytes after indicated treatments. (**C**) Detection of cell viability by CCK-8 assay in TC28 cells and primary chondrocytes after indicated treatments. (**D**) Detection of cell apoptosis by flow cytometry and statistical results in TC28 cells and primary chondrocytes after indicated treatments. *N* = 3, **P < 0.01, ***P < 0.001

To further explore the function of circCDR1as, its expression was inhibited by siRNAs in TC28 cells and primary chondrocytes (Fig. 2A). The treatment of DEX was used to induce LCPD in vitro. As shown in Fig. 2B, the level of circCDR1as was found to be dramatically stimulated by DEX in both TC28 cells and primary chondrocytes, which were then inhibited by combined siRNA treatment. In particular, the cell viability of TC28 cells and primary chondrocytes was significantly inhibited by the treatment of DEX, which was then rescued by the suppression of circCDR1as (Fig. 2C). Meanwhile, as detected by the flow cytometry, DEX treatment dramatically induced cell apoptosis in both TC28 cells and primary chondrocytes, which then got improved by knocking down of circCDR1as (Fig. 2D). On the molecular level, the expression levels of apoptosis-related genes were evaluated. As shown in Figure S1A, the mRNA level of Bax was significantly upregulated in both TC28 cells and primary chondrocytes in response to DEX treatment, and then inhibited by siRNA against circCDR1as. While the mRNA level of Bcl-2 was regulated in an opposite way compared to Bax, which was inhibited by DEX treatment and upregulated by circCDR1as inhibition. Consistently, the protein levels of Bax and Bcl-2 were altered as well, together with an obvious upregulation of cleaved-capase3 protein after DEX treatment and rescued by circCDR1as inhibition (Figure S1B). Surprisingly, the expression of miR-214-3p was shown to be suppressed by DEX and then upregulated by combined circCDR1as knocking down (Figure S1C). Moreover, the mRNA levels of osteoblast specific markers COL1A1 and RUNX2 were also detected in both TC28 cells and primary chondrocytes. As shown in Figure S3A, both of them were significantly downregulated upon DEX treatment and then rescued by circCDR1as knockdown, which further demonstrated that circCDR1as was involved in DEX-induced LCPD. In sum, these data demonstrated that inhibition of circCDR1as could alleviate the DEX-induced cell apoptosis in chondrocytes and rescued DEX-inhibited expression of miR-214-3p.

### Mir-214-3p was identified as a downstream effector of circCDR1as-mediated biological functions

**Fig. 3:**
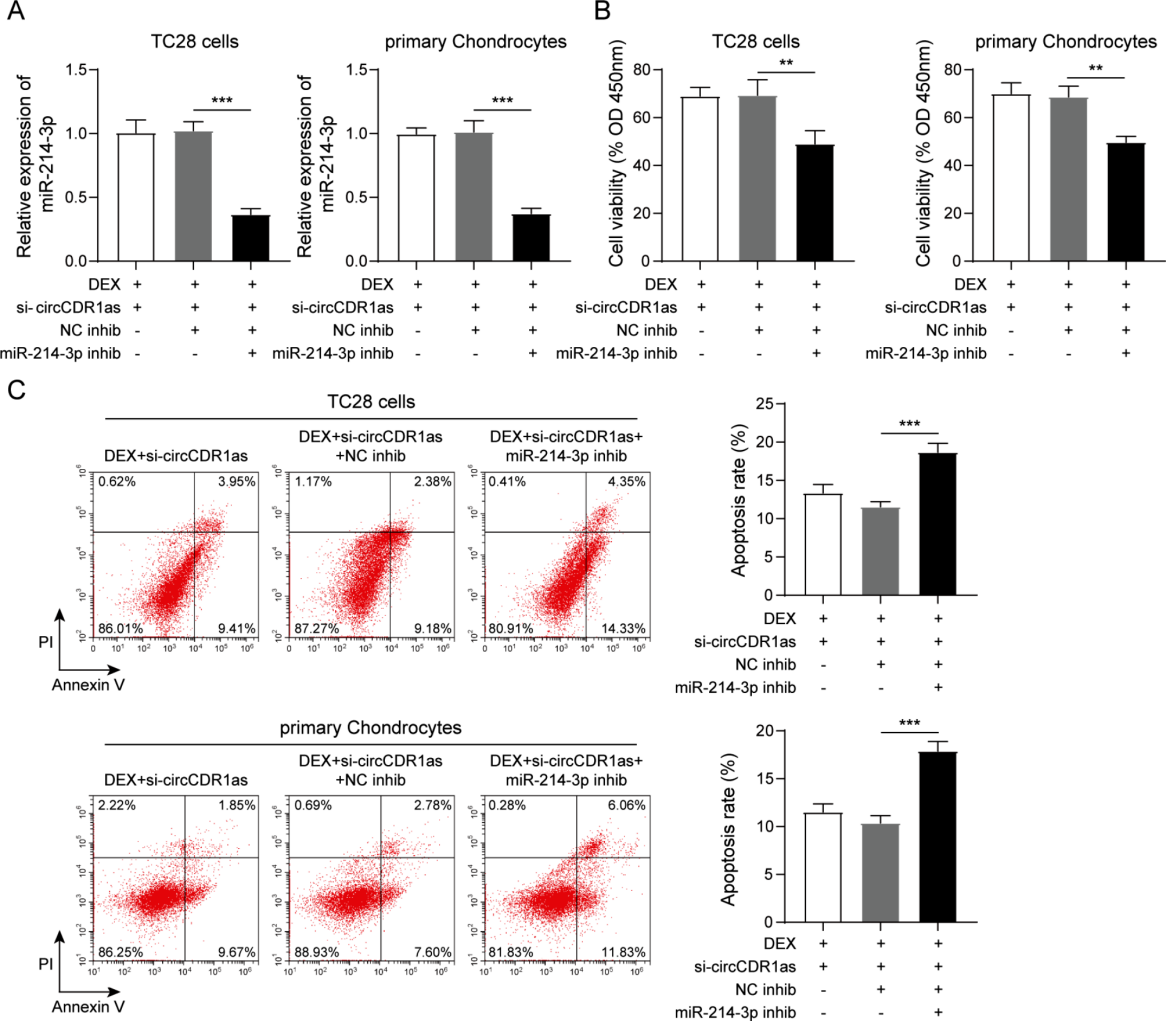
miR-214-3p was identified to be a downstream effector of circCDR1as-mediated biological functions. (**A**) Determination of miRNA inhibitor efficiency against miR-214-3p. (**B**) Detection of cell viability by CCK-8 assay in TC28 cells and primary chondrocytes after indicated treatments. (**C**) Detection of cell apoptosis by flow cytometry and statistic results in TC28 cells and primary chondrocytes after indicated treatments. *N* = 3, **P < 0.01, ***P < 0.001

As we discovered that the expression of miR-214-3p was changed by inhibition of circCDR1as, the role of miR-214-3p in circCDR1as-mediated functions was further explored. miRNA inhibitor against miR-214-3p was used to suppress its expression in both TC28 cells and primary chondrocytes combined with DEX and si-circCDR1as treatments (Fig. 3A), which was found to inhibit the cell viability and induce cell apoptosis compared to control treatment (Fig. 3B **and C**). Meanwhile, the mRNA and protein levels of Bax were significantly upregulated by miR-214-3p inhibitor, while those of Bcl-2 were downregulated (Figure S2A and B). In addition, the protein level of cleaved-caspase3 was also found to be stimulated in response to miR-214-3p inhibition compared to control treatment (Figure S2B). Meanwhile, when miR-214-3p was inhibited, the mRNA levels of COL1A1 and RUNX2 were also found to be remarkably reduced in both TC28 cells and primary chondrocytes (Figure S3B). Taken together, these results suggested that suppression of miR-214-3p could rescue the protection effect by silencing circCDR1as against cell apoptosis of chondrocytes by DEX treatment, identifying miR-214-3p as a downstream effector of circCDR1as’s biological functions.

### circCDR1as was required for the epigenetic repression of mir-214-3p by interacting with PRC2 complex

**Fig. 4:**
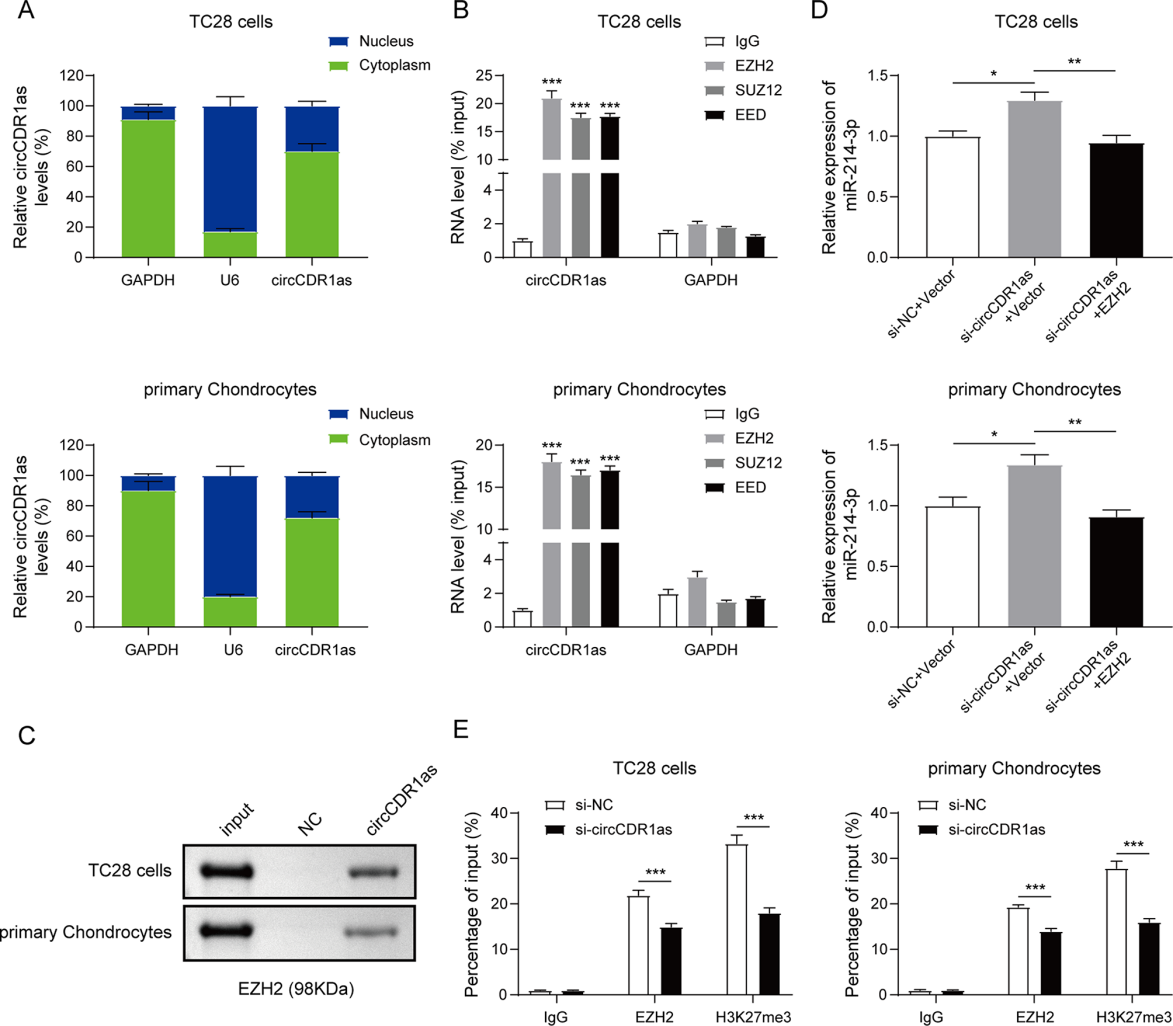
circCDR1as is required for the epigenetic repression of miR-214-3p by interacting with PRC2. (**A**) Determination of subcellular localization for circCDR1as. (**B**) Relative RNA enrichment of circCDR1as by RIP assay with EZH2, SUZ12 and EED proteins. (**C**) Detection of EZH2 protein after RNA pull-down by circCDR1as. (**D**) Detection of miR-214-3p expression in TC28 cells and primary chondrocytes after indicated treatments. (**E**) Relative signal enrichment of miR-214-3p promoter region in ChIP assay by EZH2 and H3K27me3 after indicated treatments. *N* = 3, *P < 0.05, **P < 0.01, ***P < 0.001

Since the direct binding between circCDR1as and miR-214-3p was not predicted through database, other indirect regulation potentially existed. The subcellular localization of circCDR1as in both TC28 cells and primary chondrocytes were first explored and found to be mainly located within cytoplasm (Fig. 4A). Regarding the negative correlation between the expression of circCDR1as and miR-214-3p, we speculated that circCDR1as might modulate miR-214-3p level via epigenetic repression. As one of the most important gene repressors, PRC2 complex was explored and its subunits EZH2, SUZ12 and EED were all found to directly interact with circCDR1as in TC28 cells and primary chondrocytes via RIP assay (Fig. 4B). Furthermore, RNA pull-down was used to demonstrate the association of circCDR1as and EZH2 in TC28 cells and primary chondrocytes as shown in Fig. 4C. Next, we examined if the interaction of circCDR1as and PRC2 complex was responsible for miR-214-3p expression. Knocking down of circCDR1as significantly upregulated the level of miR-214-3p in TC28 cells and primary chondrocytes, which was then inhibited by overexpression of EZH2 (Fig. 4D). Meanwhile, the occupancy of EZH2 and signal of H3K27me3 were detected in miR-214-3p’s promoter, which was significantly decreased by the inhibition of circCDR1as (Fig. 4E). Overall, these data illustrated that circCDR1as negatively regulated miR-214-3p expression via recruiting and inducing PRC2-mediated epigenetic repression.

### Silencing of circCDR1as in TC28 cells promoted M2 macrophage polarization and angiogenesis by targeting miR-214-3p

**Fig. 5:**
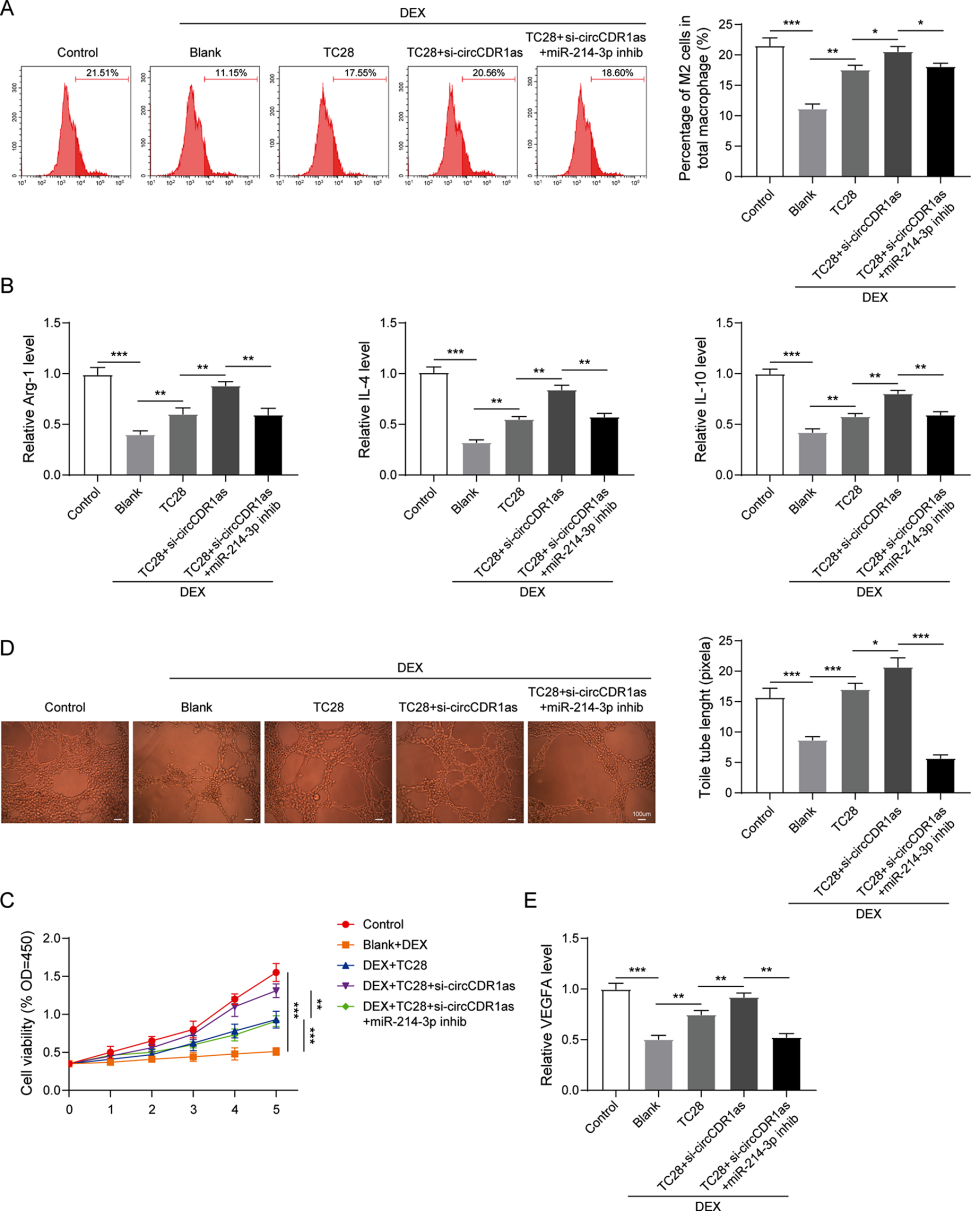
Silencing of circCDR1as in TC28 cells promoted M2 macrophage polarization and angiogenesis by targeting miR-214-3p. (**A**) Determination of M2 macrophage polarization by flow cytometry and statistic results after indicated treatments. (**B**) Detection of the expression for Arg-1, IL-4 and IL-10 in macrophages after indicated treatments. (**C**) Detection of cell viability by CCK-8 assay in HUVECs after indicated treatments. (**D**) Representative images and statistic results of tube formation ability by HUVECs after indicated treatments. (**E**) Detection of VEGFA expression in HUVECs after indicated treatments. *N* = 3, *P < 0.05, **P < 0.01, ***P < 0.001

Regarding the important roles of inflammation and angiogenesis in the development of LCPD, we then examined the effects of circCDR1as/miR-214-3p axis on M2 macrophage polarization and angiogenesis. The macrophages were obtained by isolated PBMCs followed by CD14 microbeads sorting and G-CSF stimulating. M2 polarization was induced by the treatment of IL-4 at a concentration of 20 ng/mL and characterized by the staining of CD206. As shown in Fig. 5A, M2 macrophage polarization was significantly inhibited by the treatment of DEX, which was then stimulated when cocultured with TC28 cells. When circCDR1as was suppressed in TC28 cells, the M2 macrophage polarization was further enhanced, which was rescued by the combined treatment of miR-214-3p inhibitor in TC28 cells. On the molecular level, we examined the expression levels of M2 macrophage markers Arg-1, IL-4 and IL-10. Consistent with the level of macrophage polarization, expression of these markers was suppressed by DEX treatment and enhanced by the coculture of TC28 cells. Silencing of circCDR1as in TC28 cells further increased the levels of these markers in macrophages, which were then decreased by the inhibition of miR-214-3p in TC28 cells (Fig. 5B). Meanwhile, the angiogenesis ability was also tested by evaluating the cell viability and tube formation of HUVECs. As shown in Fig. 5C and D, DEX treatment dramatically inhibited cell viability and tube formation ability of HUVECs compared to the non-treated group, which was then improved by the coculture of TC28 cells. However, inhibition of circCDR1as in TC28 cells further increased the cell viability and tube formation ability of HUVECs, which were rescued by combined treatment of miR-214-3p inhibitor in TC28 cells. Consistently, the expression of VEGFA was also changed in HUVECs, which was suppressed by DEX treatment, increased by the coculture of TC28 cells, further enhanced by si-circCDR1as treatment in TC28 cells and suppressed by miR-214-3p treatment in TC28 cells. In sum, these results strongly demonstrated that silencing of circCDR1as in TC28 cells could stimulate M2 macrophage polarization and angiogenesis via mediating the expression of miR-214-3p.

## Discussion

LCPD is an idiopathic osteonecrosis of the immature femoral head, resulting from the interrupting of blood supply in the capital femoral epiphysis and leading to the malformation of the femoral head and degeneration of the osteoarthritis due to osteonecrosis and cartilage necrosis [28, 29]. Small non-coding RNAs, such as small interfering RNAs, have exhibited promising therapeutic effects in the management of musculoskeletal-related diseases [30–32]. In the past decades, miRNAs, a class of small non-coding RNAs, have been found as critical mediators for various diseases, such as cancer, autoimmune diseases, inflammation and infertility [33]. In particular, miR-214-3p has recently been revealed to inhibit the osteogenic differentiation of human periodontal ligament stem cells by targeting ATF4 [34]. Moreover, miR-214-3p has also been reported to suppress chondrogenesis by affecting chondrocyte differentiation [20]. Most importantly, the expression of miR-214-3p has been found to decrease cell apoptosis via targeting Bax in the LCPD model, suggesting an important role of miR-214-3p in the development of LCPD [21]. In the present study, miR-214-3p was shown to be significantly downregulated in LCPD patients’ samples, which was consistent with the previous report. Intriguingly, circCDR1as was found to be significantly increased in LCPD patients’ samples compared to healthy controls, with a negative correlation to the level of miR-214-3p. This finding indicated that circCDR1as might serve a crucial role in LCPD via regulating miR-214-3p.

circRNAs have critical roles in osteonecrosis of the femoral head by mediating osteoblasts activity, endothelial cells damage and stem cells osteogenic differentiation [35]. CircCDR1as, also known as ciRS-7, is deeply involved in multiple cellular processes, including cell viability and apoptosis. For instance, circCDR1as was shown to inhibit the proliferation of bone microvascular endothelial cells by targeting miR-135b/FIH-1 axis [36]. Meanwhile, it has also been found that circCDR1as could promote cell apoptosis of hypoxia-induced mouse cardiac myocytes [37]. Additionally, circCDR1as has been identified to stimulate inflammatory responses [38]. Most importantly, increased expression of circCDR1 was found in necrosis tissue and plasma of patients with osteonecrosis of the femoral head that correlated with more pain and worse function [15]. Consistently in this work, DEX treatment was found to induce the expression of circCDR1as in both TC28 cells and primary chondrocytes, leading to a decrease in cell viability and an increase in cell apoptosis. When circCDR1as was suppressed, the DEX-induced cell damage was alleviated, strongly suggesting that the upregulation of circCDR1as was responsible for DEX-induced cell viability inhibition and apoptosis stimulation. Meanwhile, expression of osteoblast specific markers COL1A1 and RUNX2 were significantly downregulated upon DEX treatment and then upregulated when circCDR1as siRNA was added. Previous study has demonstrated that circCDR1as affects osteonecrosis of the femoral head via regulating the differentiation of bone marrow stem cells [14]. Our results for the first time identified the direct link between circCDR1as and primary chondrocytes. Regarding the negative correlation between circCDR1as and miR-214-3p in LCPD, we speculated that circCDR1as exerted its function via inhibition of miR-214-3p. Intriguingly, inhibition of miR-214-3p in DEX and si-circCDR1as treated TC28 cells or primary chondrocytes significantly suppressed cell viability, induced cell apoptosis, and downregulated the expression of COL1A1 and RUNX2, indicating that the protective effect by silencing circCDR1as against DEX treatment could be rescued by combined downregulation of miR-214-3p.

To further determine the mechanism of how circCDR1as regulated miR-214-3p, we first examined the subcellular localization of circCDR1as and found it to be mainly within the nucleus, indicating that circCDR1as might not be able to sponge miR-214-3p in the cytoplasm. Other than directly sponging miRNA, circRNAs have also been reported to interact with epigenetic regulators to modulate the expression of downstream targets. For example, circGSK3B was found to directly interacted with EZH2, blocking its binding to the RORA promoter region and upregulating the expression of RORA [39]. Consequently, we analyzed the potential interaction of circCDR1as and PRC2 complex via RIP assay and found that circCDR1as could bind into the subunits including EZH2, SUZ12 and EED, which was further validated by RNA pull-down of circCDR1as to detect EZH2 protein in both TC28 cells and primary chondrocytes. Most importantly, the binding of EZH2 and H3K27me3 modifications were detected in the promoter region of miR-214-3p, which could be decreased by silencing circCDR1as. These results for the first time demonstrated that circCDR1as regulated the expression of miR-214-3p in the context of LCPD via PRC2-mediated epigenetic suppression.

Regarding the critical roles of persistent inflammatory response and blood supply in the development of LCPD, we also assessed the impact of the circCDR1as/miR-214-3p axis on M2 macrophage polarization and angiogenesis. Consistent with previous reports [26, 40], the occurrence of LCPD, induced by DEX treatment, significantly impaired M2 macrophage polarization and angiogenesis ability, which could be rescued by the coculture with TC28 cells. Surprisingly, when circCDR1as was inhibited in TC28 cells, the M2 macrophage polarization and angiogenesis ability got further improved, which were then significantly suppressed by the treatment of miR-214-3p inhibitor in TC28 cells. These results strongly demonstrated that besides the direct influence on chondrocytes, the circCDR1as/miR-214-3p axis also played a critical role in the development of LCPD via modulating the environment, such as inflammation response and angiogenesis.

There were still some limitations in the current work that needed to be addressed in the future work. For instance, pathological results and X-rays from patient specimens, as well as the expression of miR-214-3p and circCDR1, would be needed to enhance the understanding of the disease’s pathological features. Meanwhile, further exploration of miR-214-3p expression pattern in different disease grades were necessary to demonstrate its differential expression was a linear or bidirectional change. Moreover, instead of using HUVEC cells only, pathological examinations with evaluation of the expression of vascular markers and correlation analysis in different patient specimens would contribute to a more comprehensive understanding of the role of miR-214-3p in angiogenesis. In addition, monitoring the changes in markers such as COL2A1, ACAN and MMP13 would provide a more accurate reflection of chondrocyte functional status. Given a precise answer for unlocking these questions mentioned above would provide a novel insight into LCPD therapy.

## Conclusions

In conclusion, this work revealed that highly overexpressed circCDR1as and lowly expressed miR-214-3p in LCPD patients, exhibited a negative correlation. Functional research also confirmed that loss of circCDR1as could alleviate DEX-induced cell injury in chondrocytes. Moreover, circCDR1as epigenetically suppressed miR-214-3p via recruiting PRC2 complex by H3K27me3 modification. Silencing of circCDR1as in chondrocytes also proved to stimulate M2 macrophage polarization and angiogenesis by targeting miR-214-3p. Our study illustrated the mechanism of circCDR1as in LCPD development by suppressing miR-214-3p, which may provide promising targets for LCPD treatment.

## Electronic supplementary material

Below is the link to the electronic supplementary material.

Supplementary Material 1

Supplementary Material 2

## Data Availability

All data generated or analyzed during this study are included in this published article.

## Abbreviations

3'UTR: 3’-untranslated region
ChIP: Chromatin immunoprecipitation
circRNAs: Circular RNAs
FBS: Fetal bovine serum
HCs: Healthy controls
HUVECs: Human umbilical vein endothelial cells
IP: Immunoprecipitation
LCPD: Legg-Calvé-Perthes disease
miRNAs: MicroRNAs
RIP: RNA immunoprecipitation
siRNAs: Small interfering RNAs
