# Supplementary material for: Identification of circRNA CDR1as/miR-214-3p regulatory axis in Legg-Calvé-Perthes disease
Source: Orphanet J Rare Dis. 2024 Oct 15;19:380. doi: 10.1186/s13023-024-03394-5 (PMC11481470; doi:10.1186/s13023-024-03394-5)
Supplement: Supplementary file 2 — Supplementary Material 2 [file 13023_2024_3394_MOESM2_ESM.docx]

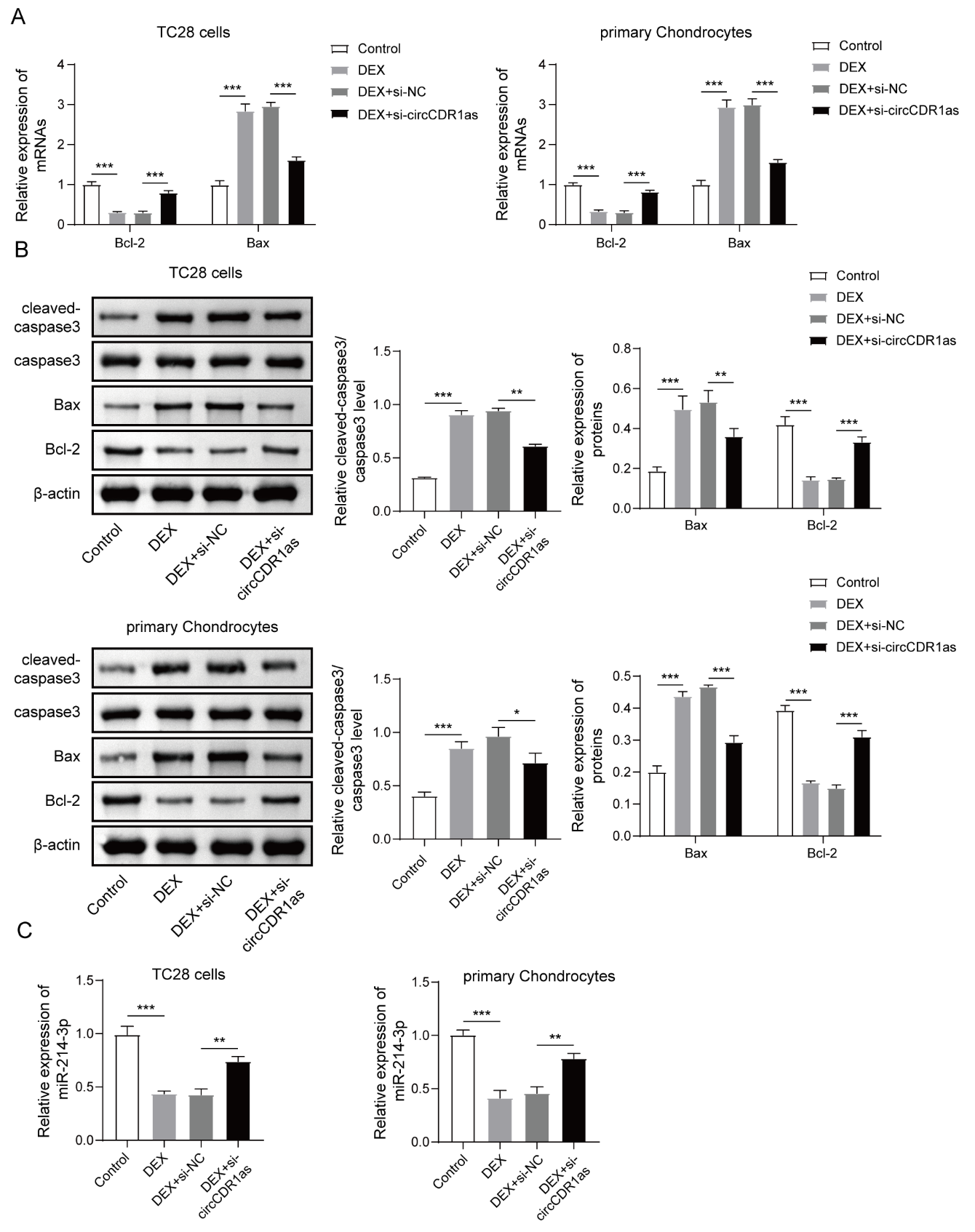


**Figure S1.** (A) mRNA and (B) protein levels of apoptosis related genes in TC28 cells and primary chondrocytes after indicated treatments. (C) Detection of miR-214-3p expression in TC28 cells and primary chondrocytes after indicated treatments. N=3, **P* < 0.05, ***P* < 0.01, ****P* < 0.001.


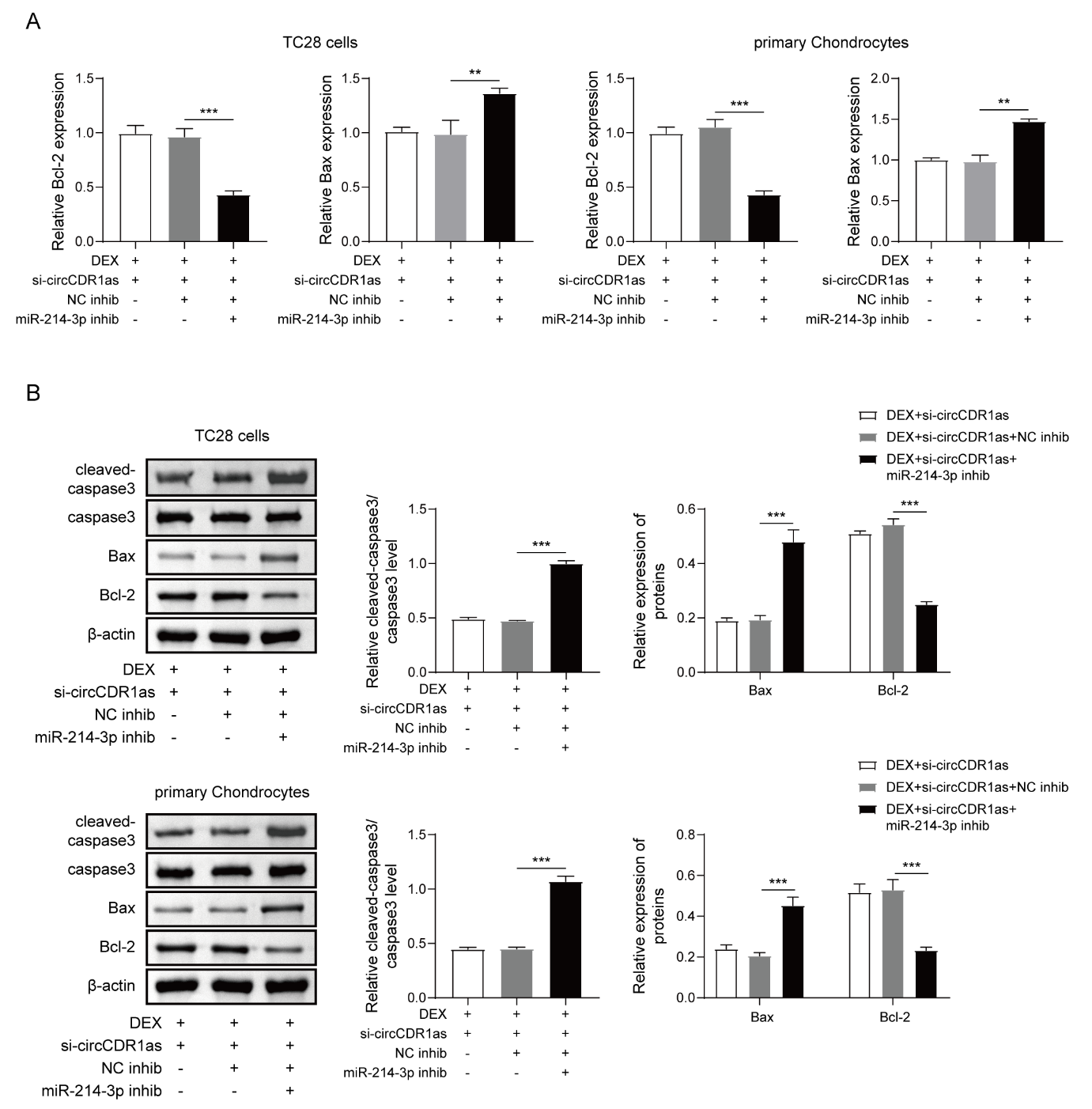


**Figure S2.** (A) mRNA and (B) protein levels of apoptosis related genes in TC28 cells and primary chondrocytes after indicated treatments. N=3, ***P* < 0.01, ****P* < 0.001.


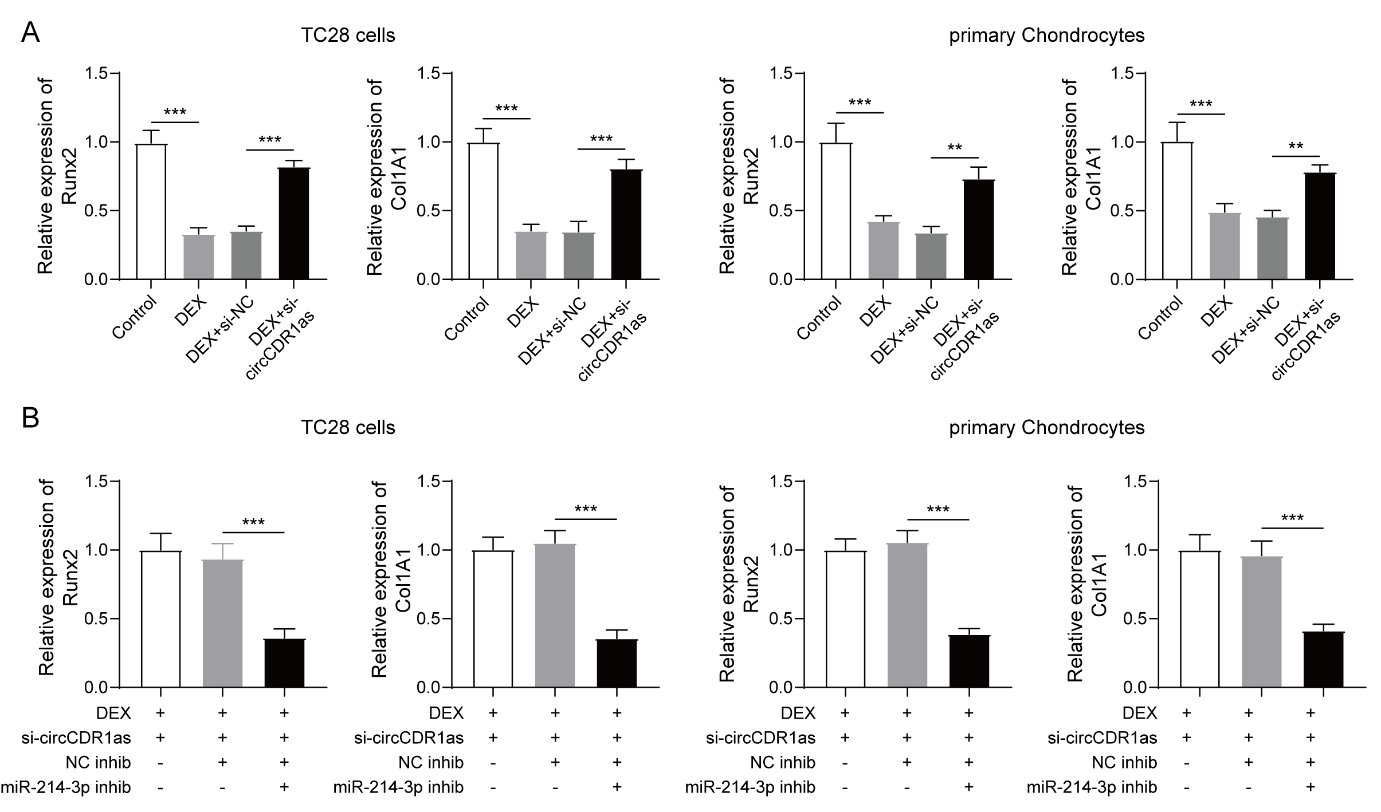


**Figure S3.** (A) Measurement of the expression of COL1A1 and RUNX2 in TC28 cells and primary chondrocytes by qRT-PCR assay. (B) Detection of the expression of COL1A1 and RUNX2 in TC28 cells and primary chondrocytes by qRT-PCR assay after indicated treatments. N=3, ***P* < 0.01, ****P* < 0.001.
