# Supplementary material for: Identification of circRNA CDR1as/miR-214-3p regulatory axis in Legg-Calvé-Perthes disease
Source: Orphanet J Rare Dis. 2024 Oct 15;19:380. doi: 10.1186/s13023-024-03394-5 (PMC11481470; doi:10.1186/s13023-024-03394-5)
Supplement: Supplementary file 1 — Supplementary Material 1 [file 13023_2024_3394_MOESM1_ESM.docx]

OJRD-D-23-01064

Identification of circRNA CDR1as/miR-214-3p regulatory axis in Legg-Calvé-Perthes disease

Xia Lan; Ronghui Yu; Jianyun Xu

Orphanet Journal of Rare Diseases

Dear Dr. Lan,

Your manuscript 'Identification of circRNA CDR1as/miR-214-3p regulatory axis in Legg-Calvé-Perthes disease' (OJRD-D-23-01064) has been assessed by our reviewers. Although it is of interest, we are unable to consider it for publication in its current form. The reviewers have raised a number of points which we believe would improve the manuscript and may allow a revised version to be published in Orphanet Journal of Rare Diseases.

Their reports, together with any other comments, are below. Please also take a moment to check our website at https://www.editorialmanager.com/ojrd/ for any additional comments that were saved as attachments.

If you are able to fully address these points, we would encourage you to submit a revised manuscript to Orphanet Journal of Rare Diseases. Once you have made the necessary corrections, please submit online at:

Your username is: lanxia0815@163.com

If you forgot your password, you can click the 'Send Login Details' link on the EM Login page at https://www.editorialmanager.com/ojrd/.

Please include a point-by-point response within the 'Response to Reviewers' box in the submission system and highlight (with 'tracked changes'/coloured/underlines/highlighted text) all changes made when revising the manuscript. Please ensure you describe additional experiments that were carried out and include a detailed rebuttal of any criticisms or requested revisions that you disagreed with. Please also ensure that your revised manuscript conforms to the journal style, which can be found in the Submission Guidelines on the journal homepage.

The due date for submitting the revised version of your article is **05 Jul 2024**.

Please note, if your manuscript is accepted you will not be able to make any changes to the authors, or order of authors, of your manuscript once the editor has accepted your manuscript for publication. If you wish to make any changes to authorship before you resubmit your revisions, please reply to this email and ask for a 'Request for change in authorship' form which should be completed by all authors (including those to be removed) and returned to this email address. Please ensure that any changes in authorship fulfil the criteria for authorship as outlined in BioMed Central's editorial policies (http://www.biomedcentral.com/about/editorialpolicies#authorship).

Once you have completed and returned the form, your request will be considered and you will be advised whether the requested changes will be allowed.

By resubmitting your manuscript you confirm that all author details on the revised version are correct, that all authors have agreed to authorship and order of authorship for this manuscript and that all authors have the appropriate permissions and rights to the reported data.

Please be aware that we may investigate, or ask your institute to investigate, any unauthorised attempts to change authorship or discrepancies in authorship between the submitted and revised versions of your manuscript.

I look forward to receiving your revised manuscript soon.

Best wishes,

Sajid Malik

Orphanet Journal of Rare Diseases

https://ojrd.biomedcentral.com/

Reviewer reports:

Reviewer #1: The article of Lan et al contributes increasing the knowledge of the pathophysiology of Legg-Calvé-Perthes disease. Although both circCDR1 and miR-214-3p were previously found to be increased in patients with osteonecrosis, Lan et al demonstrated that circCDR1regulates the expression of miR-214-3p via PRC2-mediated epigenetic suppression in patients with Legg-Calvé-Perthes disease. Silencing of circCDR1 by siRNA was able to rescue dex-induced cell viability and apoptosis in TC28 cells and primary chondrocytes and to improve the M2 macrophage polarization and angiogenesis.

From my point of view, the article is well-written and clear without redundancy. The figures add valuable information to the text.

However, there are some grammar and typo mistakes that needed to be corrected: cf.n. document attached.

**Response:** We sincerely appreciate for your precious kind reminding, and thank you for your careful review of our paper and also for pointing out these mistakes. Following your suggestion, all the grammar and typo mistakes have been revised. We also carefully checked the whole manuscript to further improve our language. Hope the modification that we have made will satisfy you and wish to get your recognition.

Reviewer #2: Dr. Xia Lan et al provides an overview to identify circRNA CDR1as/miR-214-3p regulatory axis in Legg-Calvé-Perthes disease (LCPD). The topic is very relevant and interesting, since LCPD has become an important for Bone Biology. It will be more interesting if the following comment(s) should be assessed and taken necessary actions.

Bone remodeling process is a bone renewing approach by maintaining bone strength and requires osteoblast-regulated mineralization of the bone matrix to form new bone after osteoclast-regulated resorption of old bone [Clarke B. PMID: 18988698]. Imbalance between osteoblastic differentiation and functional osteoclasts as well as during bone remodeling can lead to a pathological state reflected in systematic disruption of bone tissues, which further cause osteopenia, osteolysis or even osteonecrosis [Anesi A, PMID: 31590328]. Excessive osteoclastic activities-osteoporosis and RA, Defective osteoclast differentiation / function-osteopetrosis and Enhancement osteoblastic activities- osteosclerosis (Seeman E, PMID: 16723616). In the sense of bone homeostasis, osteoblast play an important role in bone physiology and pathology. In Legg-Calvé-Perthes' disease (LCPD), osteonecrosis is marked by the proximal femoral epiphysis, resulting in a femoral head deformity in children. Surgery is necessary to remove the affected bone called a Femoral Head Osteotomy (FHO). Osteoblast-specific Markers such as Expressions of RUNX family transcription factor 2 (RUNX2), Type-I collagen α 1 (COL1A1) (Sainz J, PMID: 10084560), ALP and miR-34a are suppressed in the femoral head in osteonecrosis (Jiang X, PMID: 33824083. FGF2 and FAM201A affect the development of osteonecrosis of the femoral head after femoral neck fracture. (Huang G, PMID: 29382571). Moreover, miR-214-3p upregulation led to elevated levels of fibrosis biomarkers, including COL1 (Hou D, PMID: 36285295). Thus it is necessary to show the relationship between the Osteoblast specific markers like COL1a1 to circRNA CDR1as/miR-214-3p regulatory axis in LCPD

**Response:** We sincerely appreciate for your suggestive comments with respect to our paper, and all authors agree with this comment and provide extra experiments and data accordingly to further support this work. As shown in Figure S3A, mRNA expression levels of osteoblast specific markers COL1A1 and RUNX2 were detected in TC28 cells and primary Chondrocytes. Both of them were significantly downregulated upon DEX treatment and then upregulated when circCDR1as siRNA was added. Meanwhile, as shown in Figure S3B, when miR-214-3p was inhibited, the mRNA levels of COL1A1 and RUNX2 were found to be significantly decreased in both TC28 cells and primary Chondrocytes. Taken together, these results further illustrated that osteoblast specific markers were affected by cirCDR1as/miR-214-3p regulatory axis in LCPD. Due to the long shipping time for the antibodies of COL1A1 and RUNX1 (applied for Western blot detection), we are unable to conduct protein level detection of these two markers. These will be included in future studies for deeper understanding of the molecular mechanisms. Thanks again for providing this valuable comment that help us better understand and illustrate the current work. Hope the modification and explanation that we have made will satisfy you, and gently wish to get your kind tolerance. Finally, we greatly prospect to get a precious chance for the publishment on your journal.

There is additional documentation related to this decision letter. To access the file(s), please click the link below. You may also login to the system and click the 'View Attachments' link in the Action column.

https://www.editorialmanager.com/ojrd/l.asp?i=372270&l=J42N4YYK
